# Self-Assembly of CsPbBr_3_ Perovskites in Micropatterned Polymeric Surfaces: Toward Luminescent Materials with Self-Cleaning Properties

**DOI:** 10.1021/acsami.2c01567

**Published:** 2022-04-19

**Authors:** Alberto S. de León, María de la Mata, Ivan R. Sanchez-Alarcon, Rafael Abargues, Sergio I. Molina

**Affiliations:** † Dpto. Ciencia de los Materiales, I. M. y Q. I., IMEYMAT, Facultad de Ciencias, Universidad de Cádiz, Campus Río San Pedro, s/n Puerto Real, Cádiz 11510, Spain; ‡ Instituto de Ciencia de los Materiales, Universitat de Valencia, Calle Catedrático José Beltrán 2, Paterna, Valencia 46980, Spain

**Keywords:** breath figures, metal halide perovskites, self-cleaning surfaces, photoluminescence, micropatterning

## Abstract

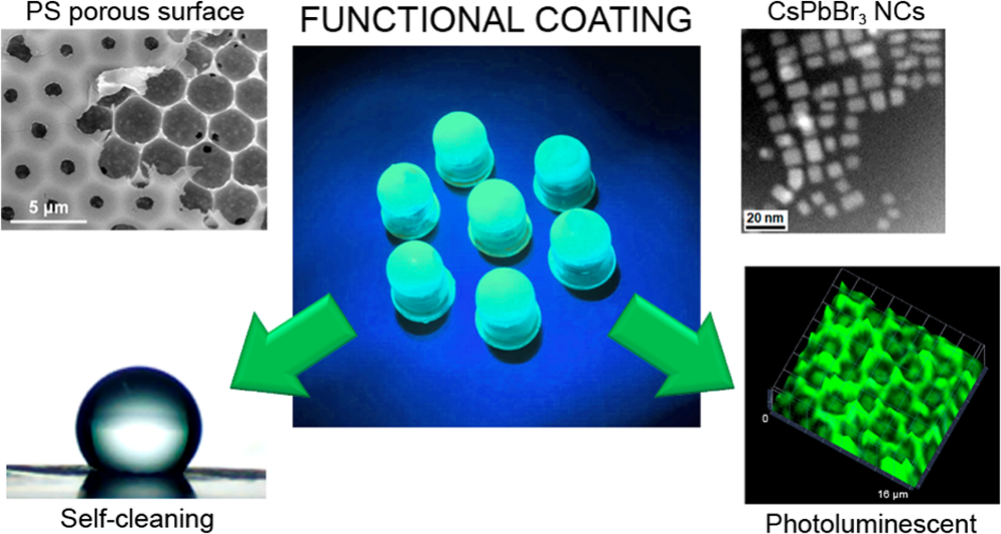

In this work, we present a series of porous, honeycomb-patterned polymer films containing CsPbBr_3_ perovskite nanocrystals as light emitters prepared by the breath figure approach. Microscopy analysis of the topography and composition of the material evidence that the CsPbBr_3_ nanocrystals are homogeneously distributed within the polymer matrix but preferably confined inside the pores due to the fabrication process. The optical properties of the CsPbBr_3_ nanocrystals remain unaltered after the film formation, proving that they are stable inside the polystyrene matrix, which protects them from degradation by environmental factors. Moreover, these surfaces present highly hydrophobic behavior due to their high porosity and defined micropatterning, which is in agreement with the Cassie–Baxter model. This is evidenced by performing a proof-of-concept coating on top of 3D-printed LED lenses, conferring the material with self-cleaning properties, while the CsPbBr_3_ nanocrystals embedded inside the polymeric matrix maintain their luminescent behavior.

## Introduction

Metal halide perovskite nanocrystals (PVK NCs) have recently gained much attention in the field of photovoltaics, semiconductor LEDs, or photodetectors because of their unique optical properties.^1^ In particular, cesium lead halide PVK NCs (CsPbX_3_, X = Br, Cl, and I) present excellent photoluminescence quantum yields (PLQY) and narrow emission line widths. Their emission spectra can be easily tuned in the visible region by changing their size or the halide atom during the synthesis step. Therefore, a large set of PVK NCs with a tunable emission can be obtained. This greatly enlarges the currently existing library of NC quantum dots.^2,3^ Regrettably, these nanomaterials tend to be unstable under environmental conditions (humidity, UV radiation, water, oxygen, heat, polar solvents...), which limits their viability in long-term applications on an industrial scale.^4,5^

Many efforts have been made to improve the stability of metal halide PVK NCs_._ For instance, the ligands present on the surface of a colloidal NC solution [typically oleylamine (OAm) and oleic acid (OA)] can be replaced by other compounds or encapsulated via surface polymerization to enhance their durability.^6−9^ Another strategy implies the use of polymeric matrixes, such as polystyrene (PS), poly (methyl methacrylate), polycaprolactone, or ethylene-vinyl acetate, where PVK NCs are embedded to form a nanocomposite. This approach has been reported to improve the stability of the NCs and their optoelectronic properties,^10−12^ while the polymer can also provide different functionalities of interest.^13^

On the other hand, PVK micropatterns are necessary for various applications such as luminescent displays. Hence, the development of new techniques for thin film formation with patterns that preserve the crystalline structures of the perovskites is required.^14^ Polymer-based nanocomposites are one of the most widespread materials employed for this purpose, typically manufactured by photolithography.^15^ However, more precise and accurate techniques have recently emerged to develop new micro-/nanopatterns with higher resolution. These include direct laser writing,^16^ focused-ion beam etching,^17^ electron beam lithography,^18^ size exclusion lithography,^19^ or 3D-printing,^20,21^ to name a few. Unfortunately, most of these techniques show a low throughput and are not suitable for rapid and low-cost production.

As an alternative to the most common patterning techniques, the breath figure approach (BF) allows the creation of honeycomb-patterned porous surfaces with controlled topography and composition in one single step. These microstructures are generated by the condensation of micron-sized water droplets on a polymeric solution in a volatile organic solvent. Because this solvent evaporates quickly, the surface temperature decreases locally, favoring the condensation of the droplets that act as a template for a honeycomb pattern. The water droplets are stabilized by the presence of amphiphilic compounds (e.g., block copolymers or surfactants), which migrate toward the interface between the water droplets and the organic polymeric solution.^22−24^ This process occurs within seconds and does not require the use of any expensive equipment, unlike top-down approaches such as lithography. Nonpolar and volatile organic solvents such as chloroform, dichloromethane, or toluene are required in BF.^25^ OA- and OAm-capped metal halide PVK NCs are soluble and stable in these solvents, so they are fully compatible with the BF approach, unlike other patterning methods that use polar solvents.^5,7^ Moreover, when the topography is optimized, the surface porosity after the BF approach can provide self-cleaning properties to the material, which is in agreement with the Cassie–Baxter model. This model considers that air bubbles remain trapped in the pores when the surface is wet, significantly increasing the hydrophobicity of the material. To achieve this, regularly distributed micro-/nanopatterns with low surface energy are required.^26−29^

Surfaces patterned by BF mostly consist of polymers, copolymers, blends, or other soft matter compounds.^28,30,31^ However, some reports show the development of hybrid materials using nanoparticles or metal ion precursors embedded in a high molecular weight polymer matrix. For instance, hybrid films for different functional applications have been prepared using Ag,^32,33^ Au,^34^ Sn,^35^ or SiO_2_.^36^

BF has also been used in the development of optical devices, including photonic crystals,^37,38^ antireflection surfaces,^39^ and photoswitchable systems.^40,41^ Different authors have developed micropatterned surfaces with luminescent properties using CdSe quantum dots.^42−44^ However, to the best of our knowledge, no research has been done so far on the fabrication of hybrid surfaces containing CsPbBr_3_ NCs by BF.

In our approach, we report on the development of a hybrid, micropatterned coating using the BF technique. This coating is composed of (1) a high molecular weight PS matrix; (2) a low molecular weight, amino-terminated PS (PS-NH_2_), which acts as a functional molecule; and (3) CsPbBr_3_ NCs capped with OAm and OA. Hybrid nanocomposites with honeycomb-patterned porous surfaces can be obtained by tuning the relative humidity (RH) and the polymer and CsPbBr_3_ NC concentrations. When these parameters are optimized, these surfaces exhibit self-cleaning properties, while maintaining their photoluminescence (PL) performance. Finally, as a proof of concept, a hydrophobic CsPbBr_3_-PS hybrid coating is generated onto 3D-printed LED lenses and tested as a luminescent down-converter, where high-energy photons are converted into low-energy photons.

## Materials and Methods

### Materials

Cesium carbonate (Cs_2_CO_3,_ 99%), 1-octadecene (ODE, 90%), OAm (80–90%), OA (90%), high molecular weight PS (*M*_w_ = 2.5 × 10^5^ g/mol), and amino-terminated polystyrene (PS-NH_2_, *M*_w_ = 5000 g/mol) were purchased from Sigma-Aldrich. Lead (II) bromide (PbBr_2_, 98+%), ethyl acetate (EtOAc), *n*-hexane, and chloroform (CHCl_3_) were purchased from Fisher Chemical. Standard stereolithography resin (clear) was purchased from XYZ printing. Isopropanol was purchased from Scharlau.

### Synthesis, Purification, and Characterization of Perovskites

The CsPbBr_3_ NCs were synthesized by the hot-injection method with some modifications.^2^ 0.32 g of Cs_2_CO_3_, 20 mL of 1-ODE, and 10 mL of OA are mixed in a 100 mL three-neck flask and heated under vacuum for 1 h at 120 °C, and the temperature is then increased to 150 °C under a N_2_ atmosphere to form a Cs-oleate precursor. On the other hand, a mixture of 1.1 g of PbBr_2_ and 40 mL of ODE is loaded into a 100 mL three-neck flask and dried under vacuum at 120 °C for 1 h. After that, 10 mL of OAm and 10 mL of OA are added under a N_2_ atmosphere, and the solution is heated to 160 °C for 1 h. Then, the solution is heated to 195 °C, and the Cs-oleate solution is swiftly injected. After 60 s, the reaction system is cooled in an ice-water bath. The CsPbBr_3_ NCs are purified by several successive centrifuging and redispersion steps with a mixture of *n*-hexane and EtOAc. Finally, the CsPbBr_3_ NCs are redispersed in CHCl_3_ with a concentration of 40 mg/mL. The absorbance spectra of the colloidal solution containing CsPbBr_3_ NCs in CHCl_3_ are measured at room temperature using a UV–visible spectrophotometer (V-770 UV–visible/NIR spectrophotometer, Jasco). PL and PLQY measurements were carried out in an FL1000 photoluminescent spectrophotometer from Edinburgh instruments using a xenon arc lamp as a light source. PL emission spectra were recorded using a wavelength of 364 nm, a dwell time of 1 s, and a wavelength step of 1 nm. PLQYs were measured with an integrating sphere module from Edinburgh Instruments with a dwell time of 1 s and wavelength step of 1 nm. Excitation and emission slits were 0,3 and 2,5, respectively. The PLQY of CsPbBr_3_ NCs was measured to be 68%.

### Fabrication of LED Shapes

A light-emitting diode (LED) lens (11.0 × 11.0 × 13.4 mm) was designed using Tinkercad online software, and the created .stl file was loaded into Nobel 1.0 stereolithography printer software. Various objects were printed using the standard XYZ resin (clear) with a layer height of either 100 or 25 μm. After printing, the objects were detached from the platform, thoroughly washed with isopropanol, and post-cured in a FormCure oven (Formlabs) using a light source of 405 nm and power of 1.25 mW/cm^2^ at 60 °C for 60 min.

### Fabrication of Porous Surfaces Via BF

**Scheme 1:**
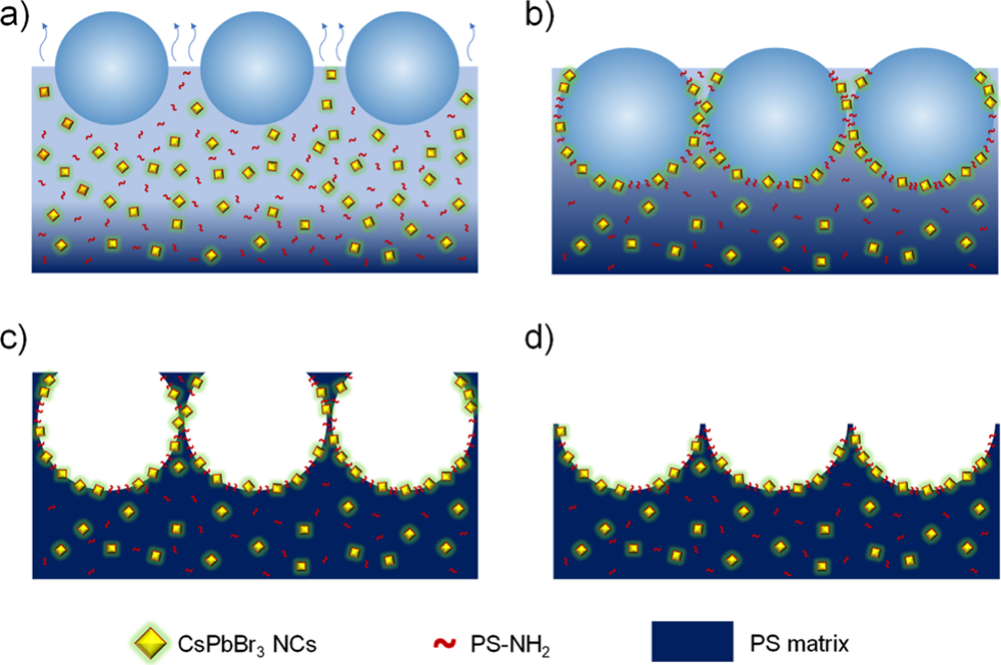
Summary of the Formation Mechanism of the Hybrid Films Via BF (a) Micron-sized water droplets start to condense in the surface of the polymeric solution caused by a local decrease of the temperature because of the fast evaporation of CHCl_3_ (endothermic process); (b) due to their amphiphilic nature, PS-NH_2_ and the CSPbBr_3_ NCs migrate towards the CHCl_3_–water interface, stabilizing the condensing water droplets in the form of a regular honeycomb pattern, avoiding their coalescence while the CHCl_3_ keeps on evaporating; (c) porous surface is formed after the CHCl_3_ and the water droplets are fully evaporated, obtaining a film with controlled topography and composition in one single step with a preferential distribution of PS-NH_2_ and CsPbBr_3_ NCs inside of the pores. The film is formed in less than 2 min; (d) a pincushion-like surface is obtained after a simple peeling process with Scotch tape.

Flat substrates (Ted Pella Inc, round glass coverslips 12 mm dia.) were coated by drop-casting 30 μL of a solution of CsPbBr_3_ NCs (1–20 wt %), PS-NH_2_ (10 wt %), and PS (70–89 wt %) in CHCl_3_ with different relative concentrations (see Table 1) inside a polycarbonate closed chamber (21.2 × 16.2 × 18.1 cm^3^) at 20 ± 1 °C and controlled RH. The RH was fixed using Petri dishes containing silica gel (RH = 20%), a saturated solution of NaNO_2_ (RH = 60%), NaCl (RH = 70%), and water (RH = 98%). Before drop-casting, the chamber was kept tightly closed until the RH reached equilibrium. Typically, at least four different surfaces were prepared to ensure the reproducibility of the results. Alternatively, nonplanar surfaces were also coated, using the previously 3D-printed LED shapes as substrates. In this case, the coating was performed by dip-coating the LED shapes for some seconds in a glass vial containing 3–5 mL of Pk10 (see Table 1) and drying them inside a closed chamber under controlled RH. This was done to achieve a more homogeneous coating on these nonplanar, larger surfaces. In all cases, the coatings are formed in less than 2 min. A detailed explanation of the formation mechanism of the porous films by BF can be found in Scheme 1.

**Table 1:** Concentration of CsPbBr_3_ and PS-NH_2_ of the Solutions Used for the Different Surfaces Prepared

	CsPbBr_3_ NCs (wt %)	PS-NH_2_ (wt %)	PS (wt %)
Pk1	1	10	89
Pk2	2	10	88
Pk5	5	10	85
Pk10	10	10	80
Pk20	20	10	70

### Characterization

Optical images of the surfaces were acquired using a Nikon MA100 inverted light microscope. Scanning electron microscopy (SEM) measurements were performed in a field-emission FEI Nova NanoSEM 450. The SEM samples, including the as-prepared and peeled surfaces, as well as transversal sections of the membranes, were Au-coated (5 nm thick) before observation. The analysis of the average pore size (*D*_p_) was performed using image analysis software ImageJ. The data were collected from different regions of each surface studied. The results were averaged, and errors were presented as the standard deviation of these measurements. Transmission electron microscopy (TEM) analyses were carried out in a Thermo Scientific Talos F200X (S)TEM microscope operated at 200 kV, working in scanning mode (STEM), and equipped with four in-column SDD Super-X detectors for energy-dispersive X-ray analysis (EDX). The as-grown hybrid porous membranes were directly transferred to TEM Cu grids for the analysis, thereby avoiding further sample preparation for planar view measurements, while a cross section of the sample was obtained by a FIB lift-out technique. Confocal laser scanning microscopy (CLSM) images were taken using a confocal Zeiss LSM 900 Airyscan 2 microscope. Images were taken using the set of filters for Alexa Fluor 488 at magnifications of 25× and 40× and analyzed using Zeiss Zen 3.3 software. Contact angle measurements were done on the porous substrates prepared on the glass coverslips using deionized water on an FDM-printed goniometer. A coupled digital microscope was used to capture the images of the water droplets, and the results were analyzed using the contact angle plugin of ImageJ software.^45^ Moreover, a test with deionized water was done to evaluate the wetting properties of the surface-treated LED lenses. For this purpose, the deionized water was previously dyed with a small amount of methylene blue for clearer observation. At least 3 droplets of 30 μL were placed on the top of the LED lenses, and their surface adhesion was video-recorded. PL and PLQY measurements were carried out in an FL1000 photoluminescent spectrophotometer from Edinburgh instruments using a xenon arc lamp as a light source.

## Results and Discussion

Highly luminescent CsPbBr_3_ NCs were synthesized by hot injection using OAm and OA as capping agents.^2^ The as-prepared NCs in the CHCl_3_ solution show an excitonic absorption band at 505 nm and PL band centered at 520 nm with a full width at half maximum ≈ 24 nm and PLQY ≈ 68% (Figure S1). The TEM image (Figure S2) shows OAm-OA-capped CsPbBr3 NCs with cubic shape and an average edge length of 12 ± 0.7 nm.

Then, different porous surfaces were fabricated using the BF technique. These hybrid films are composed of a high molecular weight PS matrix, CsPbBr_3_ NCs, and low molecular weight PS-NH_2_. PS confers good mechanical properties and stability to the film, CsPbBr_3_ NCs provide the functional properties as a light emitter, while PS-NH_2_ enhances the regularity of the pores formed. The solutions are injected inside a closed chamber with controlled RH. Under such conditions, the CHCl_3_ evaporates quickly, decreasing locally the temperature because this evaporation is an endothermic process. This causes the condensation of micron-sized water droplets on top of the surface of the polymer solution (Scheme 1a). The droplets grow, stabilized by the amphiphilic compounds (particularly PS-NH_2_), which tend to migrate toward the CHCl_3_–water interface and modify the surface tension of the system. This enables the disposition of the droplets in a well-ordered manner, avoiding their coalescence.^22^ During this process, the CsPbBr_3_ NCs are also expected to be dragged to the interface together with PS-NH_2_, given the amphiphilic nature of the OA and OAm ligands (Scheme 1b). The water droplets act as templates for the formation of porous materials and evaporate after the CHCl_3_ is totally removed and the polymeric film is formed. This allows us to obtain a hybrid, porous film with a preferential distribution of CsPbBr_3_ NCs inside the pores, typically in less than 2 min (Scheme 1c). Moreover, the porous films can be further modified by a widely used peeling protocol with Scotch tape, creating pincushion-like structures^46−49^ (Scheme 1d).

**Figure 1:**
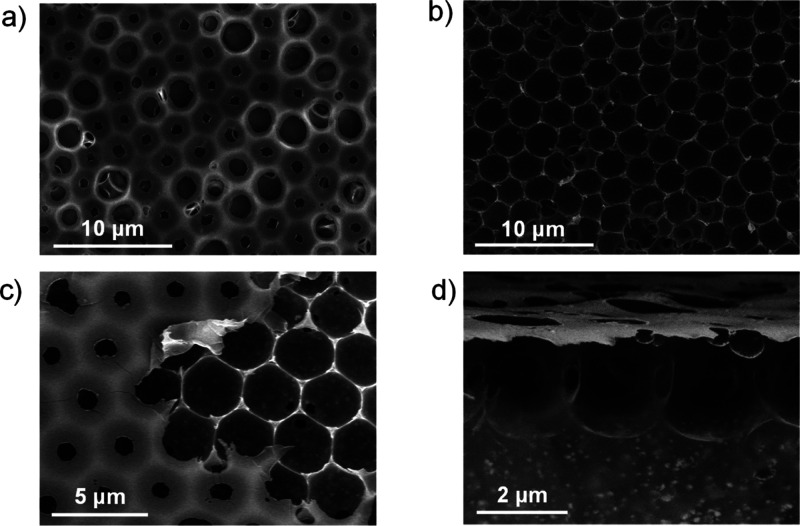
SEM images of Pk10 surfaces prepared at 98% RH (a) before and (b) after peeling; (c) top and (d) cross-sectional SEM images of Pk20.

The honeycomb-patterned films formed from Pk10 solutions are shown in Figure 1a,b, before and after peeling the top layer. Complementary images of different surfaces are shown in Figure S3, illustrating the reproducibility of these films. SEM analysis shows the homogeneous pore distribution, with pores forming a honeycomb pattern. Two different pore sizes can be differentiated before peeling (Figure 1a), while after peeling, there is an apparent unimodal pore size distribution of larger pores. This is caused by the BF mechanism. During this process, the condensing water droplets tend to sink into the polymer solution (while the CHCl_3_ is evaporating) due to surface tension effects, which can lead to pores looking smaller than they are, while creating multilayer pore structures. This fact is confirmed in Figure 1b, where some connected pores at different heights in different layers are seen. This behavior was observed for the whole range of CsPbBr_3_ NC concentrations studied (Pk1–Pk20), as shown in Figures S4, S5. The pore size does not significantly vary with the CsPbBr_3_ NC concentration, having sizes of 1.1–1.5 μm before peeling and 2.1–2.9 μm after peeling (Table S1). The average pore depth was 2.0–2.4 μm. Most probably, PS-NH_2_ is mainly responsible for stabilizing the water droplets, leading to ordered honeycomb-patterned films during the BF process.^50,51^ Additionally, in the proposed mechanism, the CsPbBr_3_ NCs are also self-driven toward the CHCl_3_–water interface due to the amphiphilic behavior of their OA and OAm ligands. This would explain why the pore size does not significantly vary in the different surfaces studied because the PS-NH_2_ concentration is always kept constant. In fact, when the surfaces are prepared in the absence of PS-NH_2_, larger pores with higher polydispersity are obtained (see Figure S6). As stated before, PS-NH_2_ is an amphiphilic molecule with a hydrophobic block (PS) of the same nature as the polymeric matrix and a hydrophilic amino end-group, which drives PS-NH_2_ toward the interface of the pores during the film formation.^50,51^ OAm, present on the surface of the CsPbBr_3_ NCs, has a similar structure to PS-NH_2,_ but its hydrophobic chain does not allow the creation of well-ordered porous structures. The effect of the RH on the formation of the pores was also investigated (see Figure S7). In agreement with the BF mechanism, lower RH results in smaller pores because less water condenses during the film formation, likely due to the smaller size of the water droplets.^22,23^ When the RH is reduced below saturation, films with a more irregular topography are observed. For 60 and 70% RH, the films present both porous and flat regions. Below 60% RH, the films obtained are flat.

Figure 1c shows the surface of sample Pk20 after peeling, illustrating both peeled and nonpeeled regions. Importantly, the SEM study was performed by using a circular back-scattered detector, rendering chemical contrast as collecting back-scattered electrons rather than secondary electrons.^52^ Thus, the small brighter features at the bottom of the pores (peeled regions) denote the presence of the CsPbBr_3_ NCs. The lack of such features at the top surface within the nonpeeled region suggests that the PS-NH_2_ is able to interact with the CsPbBr_3_ NCs to some extent, driving them toward the interface of the pores during the film formation, which is in agreement with the proposed Scheme 1. Figure 1d shows the cross section of the film from solution Pk20, revealing interconnected pores into a well-arranged monolayer at the film surface, likely due to the water droplet stabilization during the BF mechanism, preventing their coalescence. Moreover, the cross-sectional view (Figure 1d) discloses the presence of CsPbBr_3_ NC nanostructures embedded within the volume of the film, below the porous structure, probably caused by the high CsPbBr_3_ NC concentration in sample Pk20. The observed brighter structures embedded within the PS matrix show lateral sizes between 50–200 nm (significantly larger than expected for individual CsPbBr_3_ NCs, with sizes about 10–12 nm) and are homogeneously distributed throughout the whole film.

**Figure 2:**
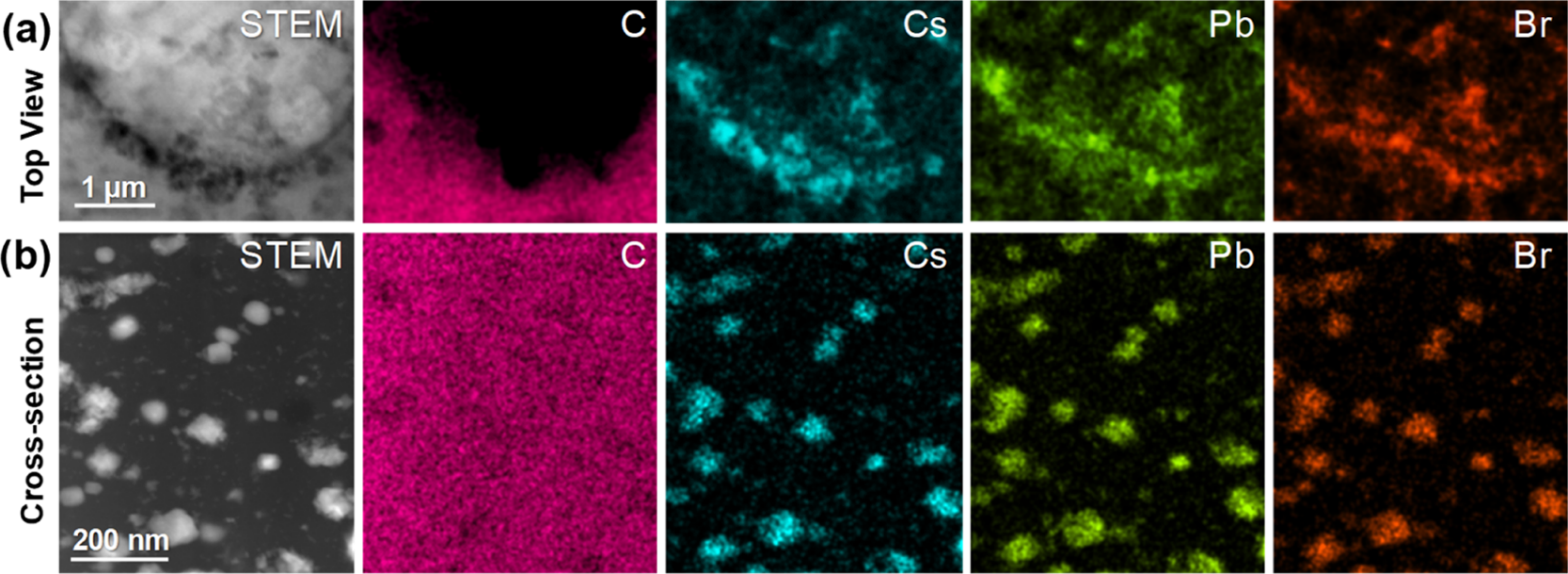
HAADF-STEM image and EDX signal maps of the cross section of Pk10 surfaces prepared at 98% RH.

In order to get deeper insights into the CsPbBr_3_ NC distribution and morphology within the PS matrix at higher resolution, the nanocomposite films were analyzed by scanning transmission electron microscopy (STEM) techniques, namely, high angle annular dark field (HAADF). Advantageously, HAADF provides *Z*-contrast images, rendering higher spatial resolution than SEM imaging. Moreover, working in STEM also allows implementing EDX analysis to assess the chemical composition for completeness of the study. Figure 2 displays a HAADF image from the top view (top) and cross-section (bottom) of the Pk10 porous film, containing several particles within the PS, whose composition has been checked by EDX. The resulting C (magenta), Cs (cyan), Pb (green), and Br (orange) EDX signal maps confirm the presence and composition of CsPbBr_3_ NCs embedded within the PS matrix, forming larger nanostructures with sizes ranging from tens to few hundreds of nm, as previously observed in Figure 1d.

**Figure 3:**
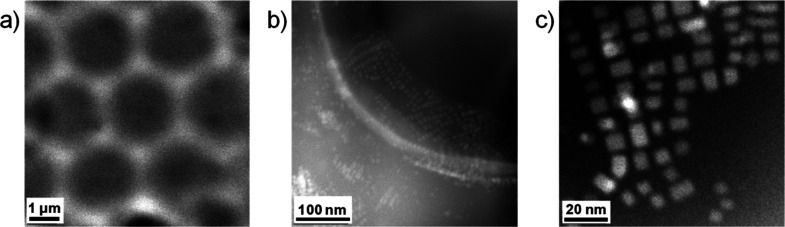
HAADF top view images of a Pk5 surface prepared at 98% RH at different magnifications, increasing from (a–c).

Once the composition of the hybrid material was confirmed, a closer inspection at the membranes from the top view was performed by HAADF in membranes prepared from Pk5 solutions at 98% RH. The honeycomb porous pattern is evident at lower magnifications (Figure 3a), while the CsPbBr_3_ NCs become visible at higher magnifications (Figure 3b,c), where the presence of highly regular nanocubes with lateral sizes of 10–12 nm is observed. This is in good agreement with the size and shape shown in the as-synthesized colloidal solution (Figure S2). Interestingly, the observed NCs self-assemble into monolayers, creating larger agglomerates, easily observed at the edges of the pores. Both OAm and OA are ligands with long aliphatic chains that provide colloidal stability and trigger the self-assembly of NCs very efficiently into supramolecular architectures of well-ordered NCs by the molecular interaction among their long alkyl groups.^53^ In fact, the interparticle spacing among CsPbBr_3_ NCs is ca. 2 nm, which corresponds to the length of both OAm and OA. Complementary images of Pk2 prepared under similar conditions are presented in Figure S8, where the distribution of the CsPbBr_3_ NCs is rather similar to that observed in Figure 3. Hence, this approach allows having a hierarchically structured material with micron-sized pores and homogeneously distributed 50–200 nm nanostructures composed of individual 10–12 nm CsPbBr_3_ NCs.

**Figure 4:**
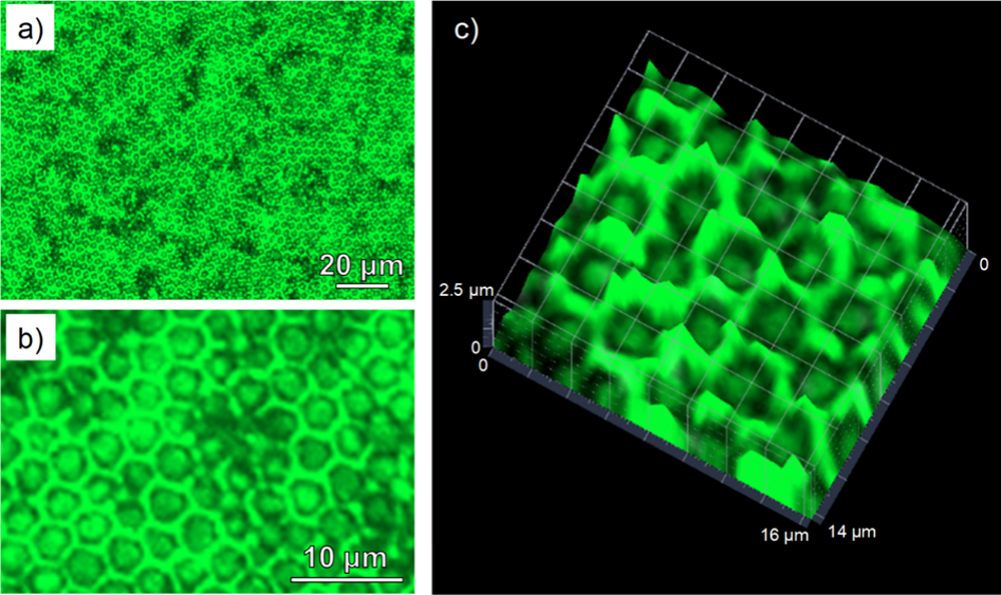
Fluorescence images of (a,b) the 2D top view of different Pk10 surfaces; (c) 3D confocal image reconstruction of a Pk10 surface prepared at 98% RH.

Thus, the BF approach has been proven as a successful methodology to obtain hybrid, porous films with a controlled distribution of CsPbBr_3_ NCs in one single step. To ensure that the perovskites maintain their functional performance (i.e., act as light emitters), CLSM studies were done. Figures 4 and S9 show the luminescent properties of different Pk10 surfaces. In general, the surfaces exhibit a homogeneous, bright green color when exposed to blue light (λ = 450 nm). The apparent lower fluorescence intensity in the bottom of the pores in the 3D-reconstruction can be attributed to scattering effects and to the fact that these surfaces are not completely even. This effect has been previously observed by us and others.^50,54,55^ In any case, these results seem to indicate that the CsPbBr_3_ NCs are homogeneously dispersed in the form of nanostructures below the diffraction limit along the porous surface, which is in agreement with previous SEM and TEM findings, and more importantly, that they preserve their PL after the film fabrication via BF. A similar effect is observed when the samples are exposed to a UV lamp, which is the principle of the down-conversion process, where high-energy photons are converted into low-energy photons with energies above the band gap. The PLQY of Pk10 porous and flat films (i.e., prepared at RH = 20 and 98%, respectively) was measured in freshly prepared samples and 1 year after fabrication to determine their long-term stability. The PLQY of flat films decreased from 24–28 to 1–2% after 1 year under ambient conditions. However, in the case of porous films, the PLQY decreased from 22–29 to 13%. Even though there is a significant decrease in the PLQY in both cases, the porous surface clearly acts as a protective layer for the CsPbBr_3_ NCs against moisture, temperature, UV light, and O_2_. As an additional control, PLQY was also measured in a sample containing 10 wt % CsPbBr_3_ in the absence of PS-NH_2_ (see Figure S6b). In this case, after 1 year, the PLQY measured was also below 2%. This confirms that the presence of PS-NH_2_ is critical for an optimized design of the porous films, contributing to the stability of the CsPbBr_3_ NCs in the PS matrix.

**Figure 5:**
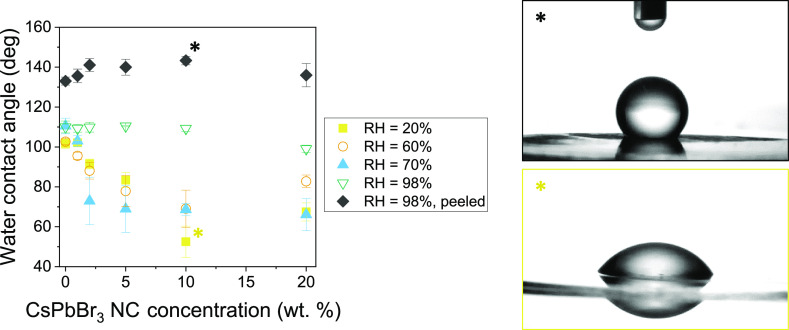
Contact angle values of the different films fabricated at 20, 60, 70, and 98% RH for CsPbBr_3_ NC concentrations ranging from 1 to 20 wt %.

The wetting properties of these films were studied by contact angle measurements. Figure 5 shows the static water contact angle values of different films prepared under different RH conditions and CsPbBr_3_ NC concentrations. The water contact angle (θ) of the samples prepared at 20, 60, and 70% RH decreases as the amount of CsPbBr_3_ NCs increases. In all these cases, from concentrations of 2 wt %, θ is below 90°, indicating that these surfaces are hydrophilic. This has probably originated from the irregularity in the formation of the pores and the presence of flat regions, as shown in Figure S7. The CsPbBr_3_ NCs on the surface, especially in the flat regions, may also increase the roughness, and therefore, the hydrophilic behavior of the films. In these cases, the porosity of the films does not enhance the hydrophobic performance of the polymeric material, and the roughness of the surface enhances the hydrophilicity of the material, as modeled by the Wenzel state.^28,30^ However, when the films are prepared under saturated RH conditions (i.e., 98%), θ increases up to ca. 120 and 140° before and after peeling, respectively. Therefore, the films prepared under these conditions have a highly hydrophobic behavior that can be used in the design of self-cleaning surfaces. Here, the wetting behavior is described by the Cassie–Baxter state, which states that bubbles of air remain trapped inside the pores when the surface is wet. This only happens when there is a defined micro-/nanopattern with high regularity. In this case, the Cassie–Baxter angle value (θ_CB_) depends on the chemistry of the material (accounted in the contact angle value of a flat film, θ_i_) and the projected void fraction of the surface *f*, as depicted in eq 1. Samples before peeling have a contact surface (i.e., projected void fraction, *f*) of 0.75–0.80, which predicts a θ_CB_ of 106–110° assuming a value of 96° for a PS flat surface.^28^ After peeling, the *f* value decreases to 0.22–0.25, which predicts an increase of the θ_CB_ up to 141–144°. These values are in good agreement with those found experimentally and with our findings in previous works. Hence, it can be concluded that these surfaces are well described by the Cassie–Baxter model. The CsPbBr_3_ NCs are not expected to play a determining role in the wetting behavior of the films after peeling because they are trapped either within the PS matrix or inside the pores. The slight increase observed in θ in this case is attributed to a slight decrease in *f*, even though the contact angle values are not significantly different for CsPbBr_3_ NC concentrations between 2 and 10 wt %. However, when the concentration is increased to 20 wt %, θ slightly decreases again. Hence, this concentration is probably high enough to keep some CsPbBr_3_ NCs present in the porous surface (even after peeling) that may participate in the wetting properties of the film, decreasing slightly the hydrophobic behavior of the coating.
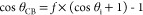
1

**Figure 6:**
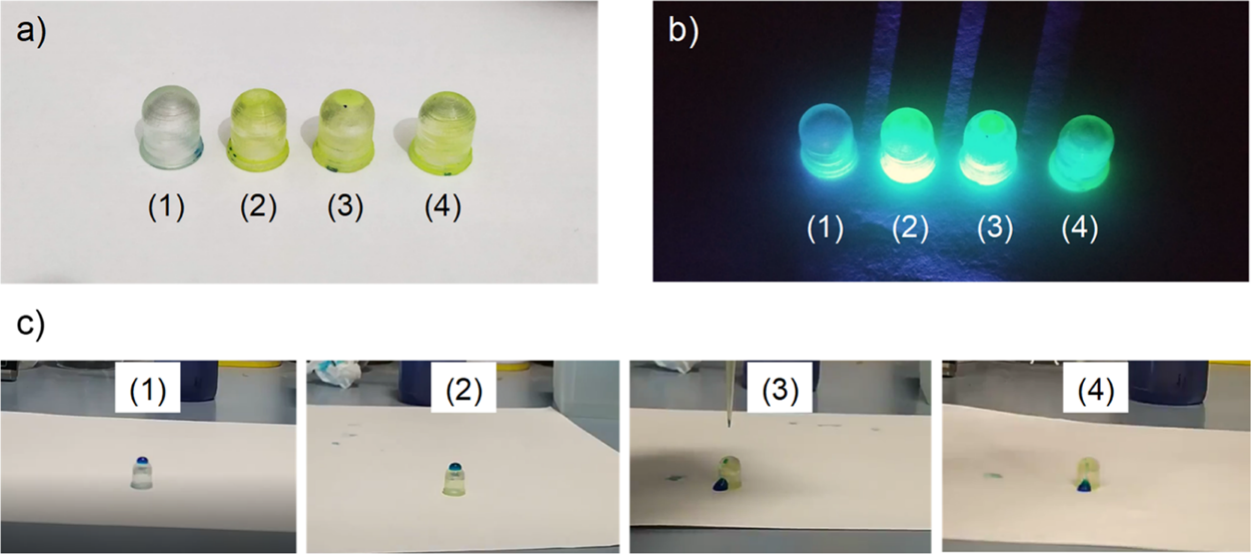
Digital images under (a) visible and (b) UV light of 3D-printed LED lenses without any treatment (1); after immersion in a Pk10 solution at 20% RH (2); after immersion in a Pk10 solution at 98% RH (3) and after immersion in a Pk10 solution at 98% RH and subsequent peeling of the surface (4); (c) digital images taken after few seconds of placing a 30 μL water droplet (dyed with methylene blue for clearer observation) on top of the 3D-printed LED lenses, evidencing their wetting behavior after treatments (1–4). The digital images in (c) were extracted from the videos available in the Supporting Information.

This approach was also tested on nonplanar surfaces. In particular, LED lenses (typically made of resin) were 3D-printed by stereolithography as proof of concept because PKV NCs are ideal candidates as light emitters for down-conversion applications. The strategy herein proposed evidences that a dual functionality can be achieved in one single step: on the one hand, the presence of CsPbBr_3_ NCs allows for a coating that makes the material luminescent. On the other hand, the formation of a controlled microstructure due to the BF mechanism makes the LED lens surface highly hydrophobic, that is, a self-cleaning surface. The procedure was done in a similar way to that of flat substrates, but the coating was done by dip-coating instead of drop-casting. This was done to achieve a more homogeneous coverage of the nonplanar surfaces. The LED lenses were immersed in a Pk10 solution inside the chamber with controlled RH. The modification of the wetting properties of these surfaces by BF is studied after three different treatments: first, BF patterning carried out at 20% RH to create a flat surface layer of CsPbBr_3_ NCs; second, BF patterning at 98% RH (saturation conditions), producing a porous thin layer containing CsPbBr_3_ NCs; and third, the same treatment at 98% RH but peeling is performed afterward to enhance the self-cleaning properties. An unmodified lens serves as the control. Figure 6a shows a digital image of the different lenses after the four different treatments. All the LED lenses coated with the CsPbBr_3_-PS nanocomposite exhibit a characteristic luminescent behavior, emitting green light upon UV irradiation (Figure 6b). It must be noted that the bare resin also shows a pale blue emission, caused by the photoinitiator of the resin. The intensity of this emission is, however, notably lower than that exhibited by the CsPbBr_3_-PS-coated LED lenses. Hence, a simple coating is enough to provide the material with their characteristic luminescent properties. The wetting behavior of the LED lenses was studied by observing the adhesion of a water droplet to the top of the curved surface of the lens. This behavior was video-recorded and is shown in the Supporting Information. Figure 6c shows the position of the droplet few seconds after its deposition on top of the LED lenses. For noncoated or coated LED lenses under low RH conditions (i.e., the coating formed is nonporous), the droplet stays on the top of the LED lens, showing high adhesion and a certain hydrophilic behavior of the material.

On the other hand, the droplet placed on the LED lenses coated at high RH conditions (i.e., 98% RH) immediately rolls down the LED lens surface and falls off, evidencing the self-cleaning behavior of these objects. Even though we previously demonstrated that the water contact angle values are higher for peeled samples, no significant differences are observed in the hydrophobicity of the surfaces before and after peeling. Thus, even though the peeling enhances the self-cleaning behavior, the key step for the successful formation of highly hydrophobic coatings is the formation of well-ordered, honeycomb porous structures via BF.

## Conclusions

We developed a new method for preparing hybrid luminescent nanocomposites for light down-conversion in LED lighting with self-cleaning properties by embedding CsPbBr_3_ NCs in a PS matrix. The topography and composition of the CsPbBr_3_-PS nanocomposites can be tuned by BF with accurate control of the resulting microstructure surface. As a result, the micropatterned nanocomposites showed an outstanding hydrophobicity without perturbing the excellent optical properties of CsPbBr_3_ NCs. Then, this strategy was also applied as a coating onto nonplanar LED lenses, validating this method on real application surfaces. The LED lenses coated by BF under high RH conditions became highly hydrophobic, exhibiting self-cleaning properties, as described by the Cassie–Baxter model. We foresee that this method can also be applied to other luminescent metal halide PVK NCs and hybrid materials in general, paving the road to multifunctional materials with self-cleaning surface properties of interest for a wide variety of applications in photovoltaics, optoelectronics, and catalysis.

## Supporting Information Available

Digital videos of the wetting behavior of the LED lenses (ZIP)
Characterization of the CsPbBr3 NCs and complementary optical microscopy, SEM, HAADF, and fluorescence microscopy images of the different porous surfaces studied (PDF)
