# Supplementary material for: Self-Assembly of CsPbBr3 Perovskites in Micropatterned Polymeric Surfaces: Toward Luminescent Materials with Self-Cleaning Properties
Source: ACS Appl Mater Interfaces. 2022 Apr 19;14(17):20023–31. doi: 10.1021/acsami.2c01567 (PMC9073833; doi:10.1021/acsami.2c01567)
Supplement: Supplementary file 2 — am2c01567_si_002.pdf [file am2c01567_si_002.pdf]

SUPPORTING INFORMATION

Self-assembly of $CsPbBr_3$ perovskites in micro-patterned polymeric surfaces: Towards luminescent materials with self-cleaning properties

Alberto S. de León[1,*], María de la Mata[1], Ivan R. Sanchez-Alarcon[2],
Rafael Abargues[2], Sergio I. Molina[1]

[1]Dpto. Ciencia de los Materiales, I. M. y Q. I., IMEYMAT, Facultad de Ciencias, Universidad de Cádiz, Campus Río San Pedro, s/n, 11510 Puerto Real (Cádiz), Spain.
[2]Instituto de Ciencia de los Materiales. Universitat de Valencia. Calle Catedrático José Beltrán 2. 46980 Paterna (Valencia) Spain.

*corresponding author: alberto.sanzdeleon@uca.es

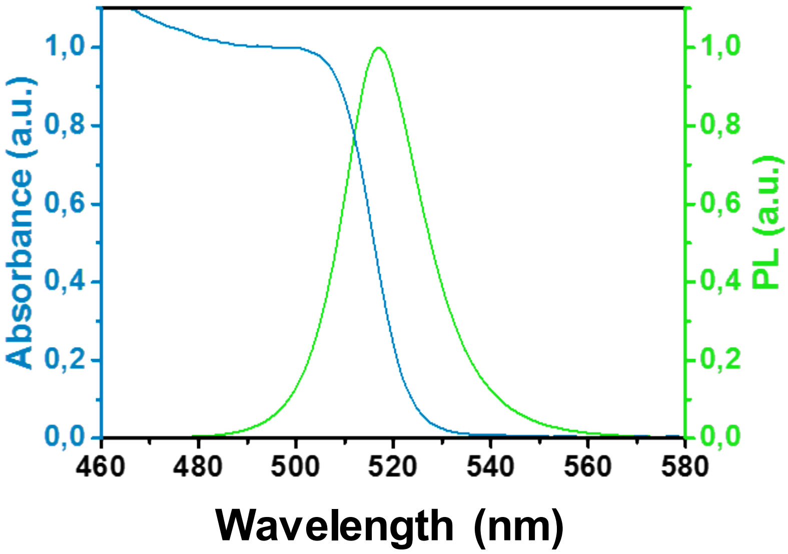

Figure S1. Absorbance and PL spectra of as-synthesized $CsPbBr_3$ NCs.

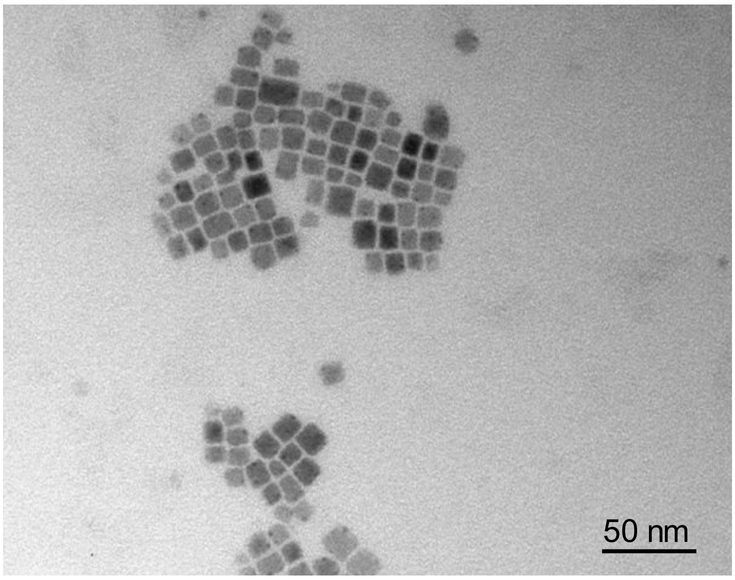

Figure S2. TEM image of as-synthesized $CsPbBr_3$ NCs.

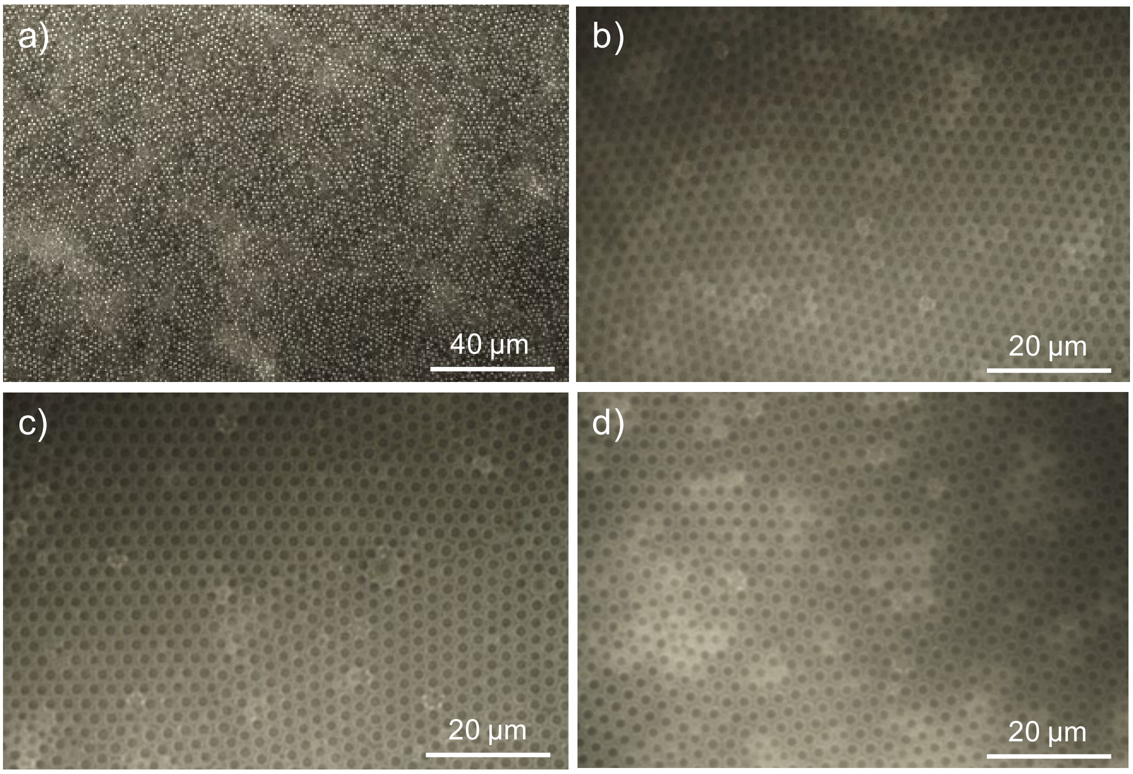

Figure S3. Optical images of different Pk10 surfaces preared at 98% RH, showing the homogeneity and repeteability of the surfaces prepared by BF.

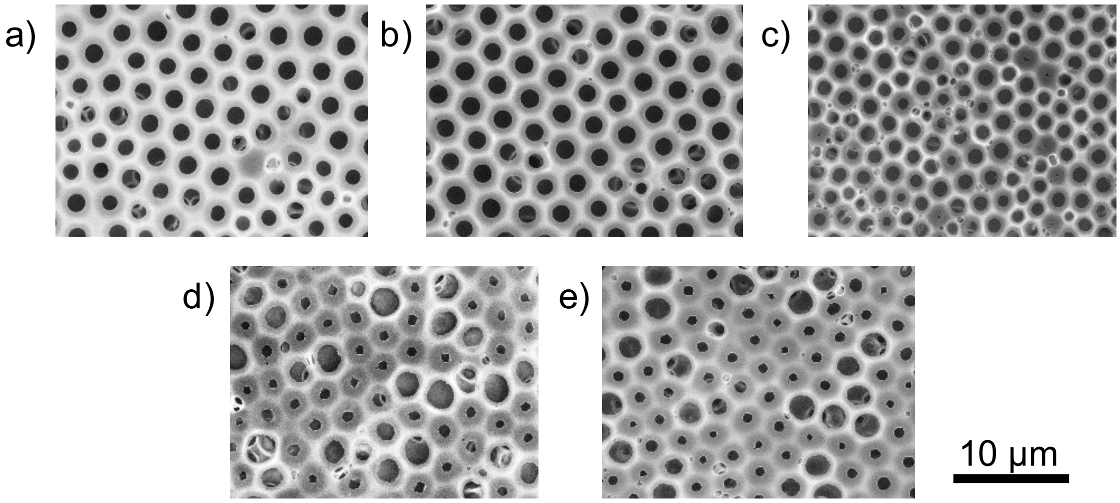

Figure S4. SE-SEM images of a) Pk1, b) Pk2, c) Pk5, d) Pk10 and e) Pk20 surfaces prepared at 98% RH before peeling. Scale bar of 10 μm is applicable to all images.

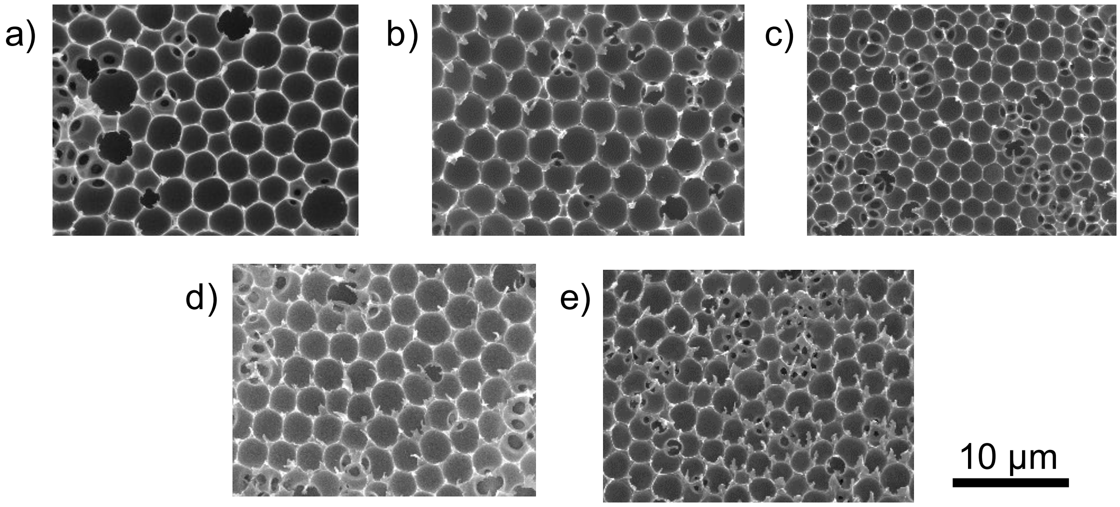

Figure S5. SE-SEM images of a) Pk1, b) Pk2, c) Pk5, d) Pk10 and e) Pk20 surfaces prepared at 98% RH after peeling. Scale bar of 10 μm is applicable to all images.

Table S1. Average pore size of the films prepared at 98% RH before and after peeling.

	**Average pore diameter size, D_p (μm)**	
	98% RH, non-peeled	98% RH, peeled
**Pk1**	1.5 ± 0.2	2.9 ± 0.3
**Pk2**	1.5 ± 0.2	2.8 ± 0.2
**Pk5**	1.1 ± 0.2	2.1 ± 0.1
**Pk10**	1.2 ± 0.6	2.7 ± 0.2
**Pk20**	1.1 ± 0.5	2.8 ± 0.1

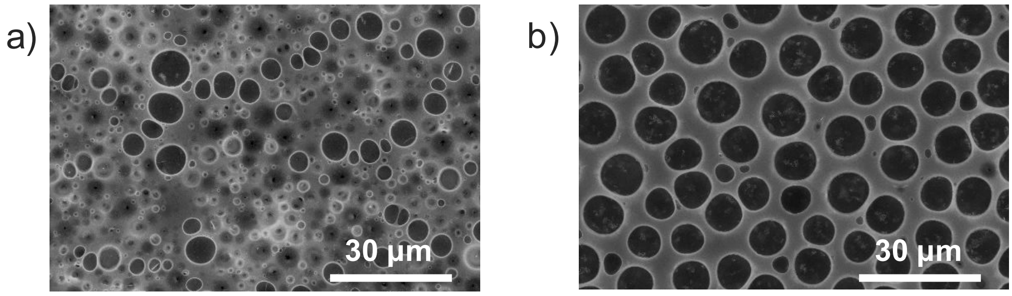

Figure S6. SE-SEM images of surfaces containing a) 5 wt% (D_p = 3.0 ± 1.8 µm) and b) 10 wt% $CsPbBr_3$ NCs (D_p = 8.1 ± 2.1 µm) prepared at 98% RH in absence of PS-NH_2. Samples were prepared from solutions in $CHCl_3$ with a total concentration of 30 mg/mL.

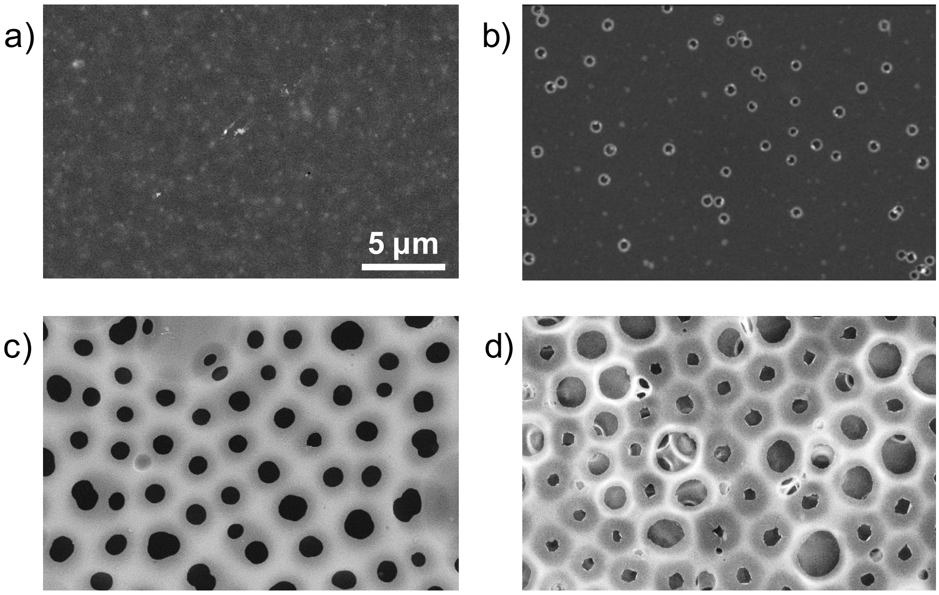

Figure S7. SEM images of Pk10 surfaces prepared at a) 20%; b) 60% (D_p = 730 ± 60 nm); c) 70% (D_p = 1.1 ± 0.2 µm) and d) 98% RH (D_p = 1.2 ± 0.6 µm). Scale bar in a) is applicable to all images.

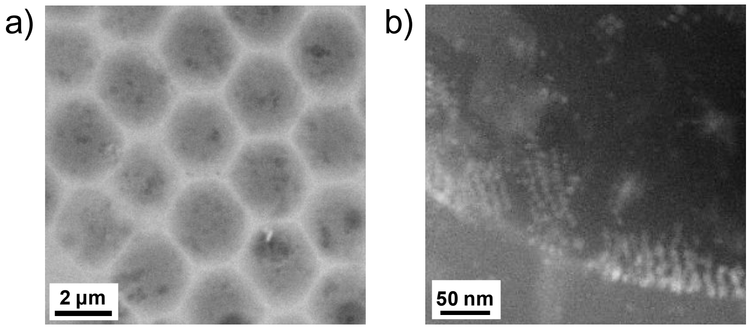

Figure S8. a,b) HAADF top view images of Pk2 surfaces prepared at 98% RH at different magnifications.

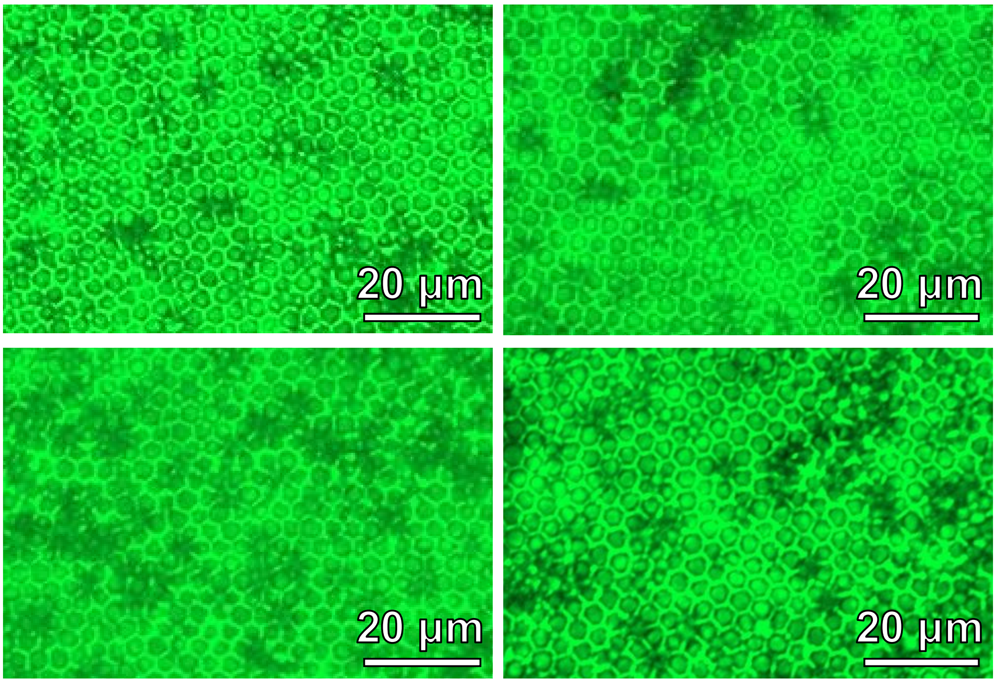

Figure S9. Fluorescence images of 2D top-view of different Pk10 surfaces prepared at 98% RH.
